# Mechanisms of HIV Entry into the CNS: Increased Sensitivity of HIV Infected CD14^+^CD16^+^ Monocytes to CCL2 and Key Roles of CCR2, JAM-A, and ALCAM in Diapedesis

**DOI:** 10.1371/journal.pone.0069270

**Published:** 2013-07-26

**Authors:** Dionna W. Williams, Tina M. Calderon, Lillie Lopez, Loreto Carvallo-Torres, Peter J. Gaskill, Eliseo A. Eugenin, Susan Morgello, Joan W. Berman

**Affiliations:** 1 Department of Pathology, the Albert Einstein College of Medicine, Bronx, New York, United States of America; 2 Public Health Research Institute, University of Medicine and Dentistry, New Jersey, Newark, New Jersey, United States of America; 3 Department of Immunology and Molecular Genetics, University of Medicine and Dentistry, New Jersey, Newark, New Jersey, United States of America; 4 Department of Neurology, Mount Sinai School of Medicine, New York, New York, United States of America; 5 Department of Neuroscience, Mount Sinai School of Medicine, New York, New York, United States of America; 6 Department of Pathology, Mount Sinai School of Medicine, New York, New York, United States of America; 7 Department of Microbiology and Immunology, the Albert Einstein College of Medicine, Bronx, New York, United States of America; University of Nebraska Medical Center, United States of America

## Abstract

As HIV infected individuals live longer, the prevalence of HIV associated neurocognitive disorders is increasing, despite successful antiretroviral therapy. CD14^+^CD16^+^ monocytes are critical to the neuropathogenesis of HIV as they promote viral seeding of the brain and establish neuroinflammation. The mechanisms by which HIV infected and uninfected monocytes cross the blood brain barrier and enter the central nervous system are not fully understood. We determined that HIV infection of CD14^+^CD16^+^ monocytes resulted in their highly increased transmigration across the blood brain barrier in response to CCL2 as compared to uninfected cells, which did not occur in the absence of the chemokine. This exuberant transmigration of HIV infected monocytes was due, at least in part, to increased CCR2 and significantly heightened sensitivity to CCL2. The entry of HIV infected and uninfected CD14^+^CD16^+^ monocytes into the brain was facilitated by significantly increased surface JAM-A, ALCAM, CD99, and PECAM-1, as compared to CD14^+^ cells that are CD16 negative. Upon HIV infection, there was an additional increase in surface JAM-A and ALCAM on CD14^+^CD16^+^ monocytes isolated from some individuals. Antibodies to ALCAM and JAM-A inhibited the transmigration of both HIV infected and uninfected CD14^+^CD16^+^ monocytes across the BBB, demonstrating their importance in facilitating monocyte transmigration and entry into the brain parenchyma. Targeting CCR2, JAM-A, and ALCAM present on CD14^+^CD16^+^ monocytes that preferentially infiltrate the CNS represents a therapeutic strategy to reduce viral seeding of the brain as well as the ongoing neuroinflammation that occurs during HIV pathogenesis.

## Introduction

HIV enters the central nervous system (CNS) soon after primary infection and results in cognitive, behavioral, and motor deficits, known as neuroAIDS or HIV associated neurocognitive disorders (HAND) [1,2]. HAND occurs in 40-70% of HIV infected individuals, despite successful combined antiretroviral therapy (cART). Its prevalence is increasing as HIV infected people live longer [3]. NeuroAIDS occurs and persists, in part, due to the ongoing transmigration of peripheral blood monocytes, both HIV infected and uninfected, across the blood brain barrier (BBB) into the CNS [4,5]. Virus is released as the infected monocytes enter the brain parenchyma, which may then infect additional CNS cells [6,7]. Infected and activated CNS cells produce neurotoxic host and viral factors, and chemokines and cytokines. These establish inflammation in the brain and lead to the neuronal damage and loss associated with HAND [8]. Even in the context of cART, low-level, chronic neuroinflammation persists in HIV infected individuals [9].

Peripheral blood monocytes are heterogeneous and subpopulations exist with distinct functions and degrees of maturation/activation. Surface markers to distinguish among human monocyte subsets include CD14, the LPS receptor, and CD16, the F_C_γIII receptor [10]. Most circulating monocytes express only CD14 and are CD14^+^CD16^-^. This population comprises 90-95% of peripheral blood monocytes. Monocytes that also express surface CD16 represent only a small percentage of monocytes in healthy individuals and are believed to be a more mature population [11].

This CD14^+^CD16^+^ population, that represents 5-10% of circulating monocytes, is increased in the blood of HIV infected individuals [12,13]. These monocytes are critical to HIV neuropathogenesis as they are highly susceptible to HIV infection, are found in the brains of HIV infected individuals with neuroAIDS, and may be predictive of the cognitive decline that occurs with HAND [14–16]. As this mature monocyte subset is present in such low numbers in healthy individuals, we developed a culture system that provides enough CD14^+^CD16^+^ cells for analyses [17].

Prior to entering the CNS, monocytes must be directed to their site of entry at the BBB by chemokines. CCL2 is a monocyte chemoattractant highly elevated in the CSF of HIV infected people with cognitive decline [18,19]. CCL2 remains increased even with successful cART [20,21]. CCR2, the only known receptor for CCL2 on monocytes [22], is implicated in many diseases characterized by chronic inflammation and infiltrating monocytes. CCR2 expression on the CD14^+^CD16^+^ population and its role in monocyte entry into the CNS during HIV infection had not been extensively studied previously.

The mechanisms by which HIV infected CD14^+^CD16^+^ monocytes cross the BBB and infiltrate the CNS are not well understood. Studies from the peripheral vasculature suggest that monocyte diapedesis is facilitated by tight junction proteins and adhesion molecules, collectively termed junctional proteins, including junctional adhesion molecule-A (JAM-A), activated leukocyte cell adhesion molecule (ALCAM), CD99, and platelet endothelial cell adhesion molecule 1 (PECAM-1) [23–27]. The homotypic interactions between junctional proteins on the monocyte and brain microvascular endothelial cells (BMVEC) of the BBB shepherd the monocyte into the brain in a zipper-like fashion which, during normal immune surveillance, does not disrupt barrier integrity [23].

JAM-A, ALCAM, CD99, and PECAM-1 are present on monocytes, but the extent to which each monocyte population expresses them and their role in HIV CNS infection is unknown [28–31]. Additionally, these junctional proteins have been extensively characterized in the peripheral vasculature, but little is known about their presence on the specialized BMVEC of the BBB. We proposed that the homophilic interactions between the junctional proteins on monocytes and the BMVEC are necessary for the well-coordinated entry of the CD14^+^CD16^+^ monocyte population into the brain and dysregulation of these proteins will promote CNS disease.

In this study, we examined the effects of HIV infection on the transmigration of CD14^+^CD16^+^ monocytes across the BBB, demonstrated that heightened sensitivity to CCL2 and increased CCR2 are a mechanism by which HIV infected cells enter the CNS, and identified cell surface markers specific to this mature monocyte subpopulation which facilitate their diapedesis. We characterized monocytes isolated from HIV(+) individuals for surface CD14 and CD16 and compared them to the monocytes cultured nonadherently in our *in vitro* system. We found that the CD14^+^CD16^+^ monocyte population that is increased in HIV(+) people closely resembled the mature monocytes cultured in our system. We determined that HIV infected CD14^+^CD16^+^ monocytes transmigrated across the BBB in far greater numbers in response to CCL2 than did their uninfected counterparts. This increase in transmigration occurred even with extremely low levels of HIV infection, similar to those seen with cART. This suggests that even with successful therapy, HIV infected monocytes may continually enter the CNS. We found this was due, at least in part, to a heightened sensitivity to CCL2 that we demonstrated was mediated by increased CCR2. We characterized the junctional proteins on CD14^+^CD16^+^ monocytes that may facilitate transmigration across the BBB. We showed that JAM-A, ALCAM, PECAM-1, and CD99 are highly expressed on CD14^+^CD16^+^ cells. We found that JAM-A and ALCAM are critical for transmigration of CD14^+^CD16^+^ monocytes across the BBB, as antibodies to these proteins inhibited their transmigration in response to CCL2.

## Methods

### Cell Isolation and Culture

Blood was obtained from HIV seronegative (HIV(-)) people or from leukopaks from the New York Blood Center according to established protocols at the Albert Einstein College of Medicine. Peripheral blood mononuclear cells (PBMC) were isolated by Ficoll-Paque PLUS (GE Healthcare, Uppsala, Sweden) density gradient centrifugation. Monocytes were isolated from PBMC by magnetic bead positive selection using the CD14 EasySep separation kit (Stem Cell Technologies, Vancouver, Canada). Monocyte purity was assessed by flow cytometry using antibodies specific for CD14 (clone M5E2), CD19 (clone HIB19), CD3 (clone HIT3a), and CD56 (clone B159) (BD Biosciences, San Jose, CA). Monocytes were >90% pure as determined by flow cytometry and contained <2% CD19^+^ B Cells, <3% CD3^+^ T Cells, and <1% CD56^+^ NK Cells. Freshly isolated “Day 0” CD14^+^ monocytes were cultured nonadherently in Teflon flasks at 2x10^6^ cells/mL for 3 days with 10 ng/mL M-CSF (Peprotech, Rocky Hill, NJ) in supplemented RPMI as previously described to facilitate monocyte maturation and yield “Day 3” monocytes that are highly enriched for CD14^+^CD16^+^ cells [17,23].

Blood from five, cART-treated HIV seropositive (HIV(+)) individuals was obtained from the Manhattan HIV Brain Bank (MHBB; U01MH083501), a research resource operating at the Mount Sinai School of Medicine in New York City, NY. Patients gave written, informed consent for the provision of blood for the purposes of HIV research. The protocol under which these samples were obtained is approved by the Mount Sinai Program for the Protection of Human Subjects Institutional Review Board. Patient demographics and virologic characteristics are in Table 1. PBMC were isolated within 2 hours of blood draw and phenotyped by flow cytometry.

**Table 1:** Demographic and immunovirologic characteristics of HIV infected participants (n=5), all of whom are on cART.

Age, years	
- Mean ± SD	53 ± 5
- Median (range)	52 (48-59)
Gender, %	
- Male	3 (60%)
- Female	2 (40%)
CD4 T-cell count, cells/Μl	
- Mean ± SD	468 ± 324
- Median (range)	475 (143-920)
Plasma HIV RNA, log copies/mL	
- Mean ± SD	2.69 ±1.62
- Median (range)	1.98 (1.30-4.46)
- Undetectable, %	2 (40%)

### Flow Cytometry

PBMC or monocyte cell surface markers were analyzed by flow cytometry using fluorochrome coupled monoclonal antibodies specific for human CD14 (clone M5E2), CD16 (clone 3G8), ALCAM (clone 3A6), JAM-A (clone M. Ab. F11), CD99 (clone TU12), PECAM-1 (clone WM59), and CCR2 (clone 48607) or corresponding isotype matched negative control antibodies (all from BD Biosciences, except CCR2, R&D Systems, Minneapolis, MN). Antibodies were titrated to determine optimal staining conditions. Cells (1-3x10^5^) were incubated in the dark on ice for 30 minutes with the appropriate antibody, washed once, and fixed with 2% paraformaldehyde. For CCR2, four separate tubes of 2.5x10^4^ cells were immunostained with anti-CD14-PE, anti-CD16-PE-Cy7, and anti-CCR2-APC (1:3 dilution) in a total volume of 15 µL and the cells from each tube were pooled before fixing. At least 10,000 events (for CD14^+^ isolated monocytes) or 60,000 events (for PBMC) were acquired with a BD Canto II flow cytometer and analyzed using FlowJo software (TreeStar v. 9.5.3, Ashland, OR).

### HIV Infection

After three days of nonadherent culture, monocytes were infected with HIV as previously described [17]. Briefly, monocytes were inoculated with 20 ng/mL HIV_ADA_ or remained uninfected. After 24 hours the virus was removed by centrifugation and the monocytes resuspended at 2x10^6^ cells/mL in fresh culture media with 10 ng/mL M-CSF in Teflon flasks. Uninfected or HIV infected monocytes were cultured for an additional two days to facilitate HIV infection and replication. Supernatant levels of HIV p24 were measured using the high-sensitivity p24 AlphaLISA (PerkinElmer, Waltham, MA).

### *In Vitro* Human BBB Model

The BBB model consists of human BMVEC (Applied Cell Biology Research Institute, Kirkland, WA) and astrocytes isolated from human cortical tissue as a part of an ongoing approved research protocol at the Albert Einstein College of Medicine. BMVEC and astrocytes were cocultured on opposite sides of a gelatin coated tissue culture insert with 3 µm pores (BD Falcon, Franklin Lakes, NJ), as previously described [32–35]. Briefly, the astrocytes were seeded on the underside of the insert and the BMVEC were added to the upper side. The cocultures were grown to confluence for 3 days, at which time the astrocytic foot processes penetrated the insert and contacted the BMVEC to seal the barrier.

### Assay of Monocyte Transmigration Across the BBB Model

Uninfected or HIV infected monocytes (2x10^5^) were added to the top of the coculture insert and transmigrated to media alone or to 200 ng/mL CCL2 (R&D Systems) for 24 hours. Each transmigration condition was performed with 4 replicate cocultures. For blocking experiments, 20 µg/mL blocking antibody to JAM-A (clone J10.4, Santa Cruz Biotechnology, Santa Cruz, CA), ALCAM (clone 81, Antigenix America, Huntington Station, NY), or purified mouse IgG1 (MP Biomedical, Solon, OH) were added to the top of the cocultures concurrently with the monocytes. After 24 hours the transmigrated cells were collected from the bottom of the chamber, immunostained with anti-CD14 APC and anti-CD16 PE-Cy7, and quantified by flow cytometry. Kinetic analysis and CCL2 dose–response was performed to determine the appropriate duration of time and optimal chemokine concentration in which to perform the transmigration assays.

### Chemotaxis Assay

Monocyte chemotaxis was evaluated using a microchemotaxis apparatus (Neuro Probe, Inc., Gaithersburg, MD). Vehicle or 1, 10, 25, 50, 100, 200, or 500 ng/mL CCL2 were added to the lower compartment of the apparatus followed by the polycarbonate filter with 5 µm pores. Uninfected or HIV infected monocytes (1x10^5^) cultured in our *in vitro* system were added to the upper compartment and allowed to migrate across the membrane for 1 hour at 37°C, 5% CO_2_. After chemotaxis, the membrane was removed, fixed, and stained with Diff-Quick. Monocyte chemotaxis was quantified by densitometric analysis of the polycarbonate membrane using UN-SCAN-IT gel digitizing software (Silk Scientific, Orem, Utah) [36].

### Western Blot Analysis

Uninfected or HIV infected monocytes from our culture system were lysed with RIPA buffer (Cell Signaling, Beverly, MA) containing protease inhibitors (Cell Signaling), and 60 µg of protein were electrophoresed on a 4-12% polyacrylamide gel (Bio-Rad), and transferred to nitrocellulose membranes. Membranes were probed with rabbit monoclonal antibodies to CCR2 (Abcam, Cambridge, MA) or GAPDH (Cell Signaling). Densitometric analysis was performed using Un-SCAN-IT gel digitizing software.

### Quantitative RT-PCR

Total RNA was extracted from monocytes using TRIzol (Invitrogen, Carlsbad, CA) and cDNA synthesis performed using 2 µg of total RNA with the iScript cDNA Synthesis Kit (Bio-Rad, Hercules, CA). Relative mRNA expression of JAM-A, ALCAM, CD99, PECAM-1, GAPDH, and β-actin was assessed by quantitative RT-PCR using the StepOne Plus Thermocycler (Applied Biosystems, Foster City, CA) Fast Cycle program (denaturation for 15 min at 95°C and 40 cycles of denaturation, 15 s at 95°C, anneal, 30 s at 60°C, and amplification, 30 s at 72°C). The ΔΔCT was calculated as previously described [37]. Oligonucleotide primers used for gene amplification are in Table 2. Single-product amplification was confirmed by melting curve analysis and primer efficiency was near 100% in all experiments.

**Table 2:** Oligonucleotide primers used to determine mRNA expression of junctional proteins by qRT-PCR.

**Gene**	**Forward Primer (5’–3’)**	**Reverse Primer (5’–3’)**
β-Actin	CTCTTCCAGCCTTCCTTCCT	AGCACTGTGTTGGCGTACAG
GAPDH	GAGAAGTATGACAACAGCCTCAA	AGTCCTTCCACGATACCAAAG
JAM-A	GTGCCTTCAGCAACTCTTCC	ACCAGATGCCAAAAACCAAG
ALCAM	CGCAATGCAACAGGAGACTA	GGCTAGATCGAAGCCTGATG
CD99	CTGGGCGGATGATGTTTACT	TCGATGGACACGTGATTTGT
PECAM-1	GATAATTGCCATTCCCATGC	TTGCAGCACAATGTCCTCTC

### Statistical Analysis

Statistical analyses were performed using Prism 5.0 software (GraphPad Software, Inc., San Diego, CA). Wilcoxon Signed Rank test or a Two-Tailed Paired T test was used to determine statistical significance (p≤0.05).

## Results

### Monocytes Matured in our *In Vitro* System Closely Resemble the CD14^+^CD16^+^ Monocytes that are Increased in HIV Infected Individuals

**Figure 1:**
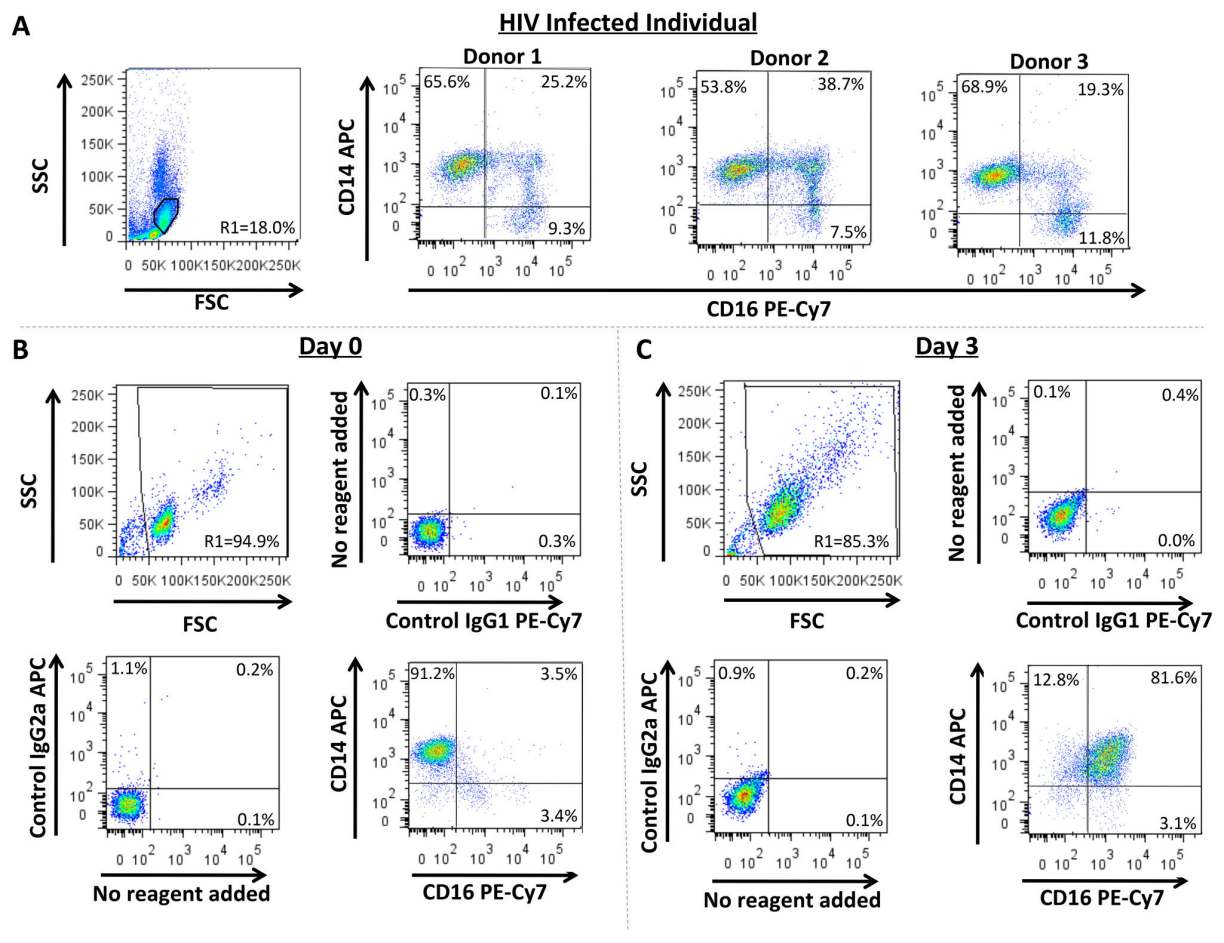
CD14^+^CD16^+^ Monocytes Increase in the Peripheral Blood of HIV(+) People and in Culture. (**A**) PBMC from HIV(+) individuals were stained with CD14 APC and CD16 PE-Cy7-coupled antibodies. Forward and side scatter were used to determine monocyte gating. The CD14^+^CD16^+^ population represented a significant percentage of monocytes in HIV infected individuals as compared to uninfected individuals (see below). Data from 3 separate donors, representative of 5, are shown. (**B**–**C**) Monocytes from HIV(-) people were isolated from PBMC using CD14 magnetic beads and analyzed by flow cytometry. Data from a single donor representative of 57 independent individuals are shown. (**B**) Freshly isolated “Day 0” and (**C**) “Day 3” monocytes matured for 3 days in nonadherent culture with 10 ng/mL M-CSF were stained with isotype matched negative control antibodies or with CD14 APC and CD16 PE-Cy7-coupled antibodies. CD14^+^CD16^+^ monocytes represented only a small percentage of freshly isolated monocytes (**B**). Our culture system significantly increased their numbers (**C**) and modeled the expansion of the CD14^+^CD16^+^ population in HIV(+) individuals (**A**).

The CD14^+^CD16^+^ monocyte subset is critical to the pathogenesis of HAND [15–17]. To examine this subset, we phenotyped circulating monocytes isolated from 5 HIV infected individuals on cART. Demographics and virologic characteristics are in Table **1**. We found high numbers of CD14^+^CD16^+^ monocytes in the peripheral blood of these individuals (Figure 1A), despite antiretroviral therapy.

To study this CD14^+^CD16^+^ population in monocytes derived from HIV(-) individuals, we previously developed a monocyte maturation culture system to enrich for these cells [17]. We found approximately 90-95% of freshly isolated monocytes from HIV(-) donors were CD14^+^ (Figure 1B). Only 5-10% of these cells were CD14^+^CD16^+^ (Figure 1B). After three days of nonadherent culture with M-CSF there was an enrichment of CD14^+^CD16^+^ monocytes (Figure 1C) and this population comprised 70 ± 16% (n=57, p<0.0001) of the cells. Thus, our tissue culture system models the expansion of CD14^+^CD16^+^ monocytes that occurs in the peripheral blood of HIV(+) individuals and provides enrichment of a monocyte population present in such few numbers in uninfected people that analyses would otherwise be difficult.

### HIV Infection Increases CCL2-Mediated Monocyte Transmigration Across the Human BBB

**Figure 2:**
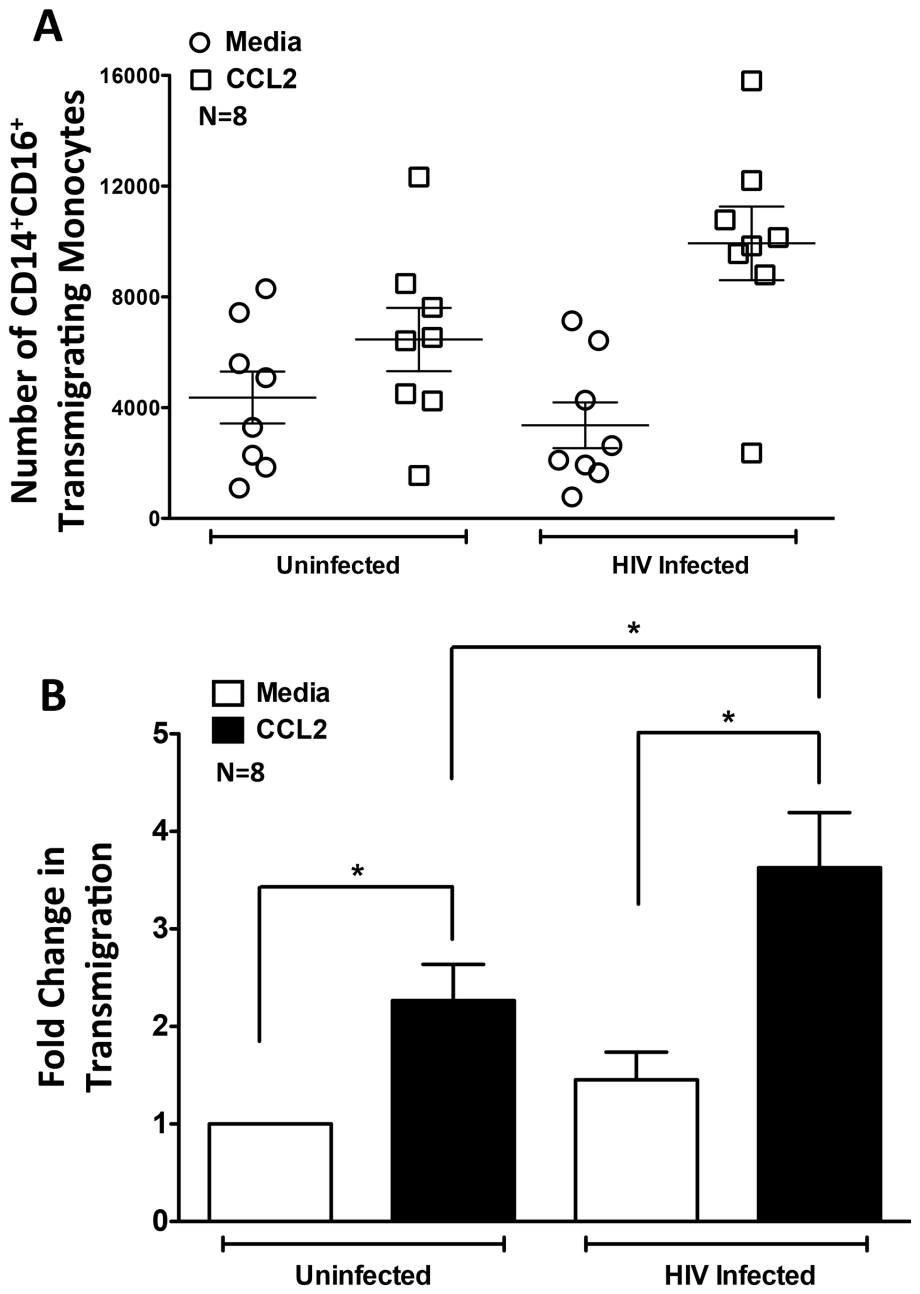
HIV Infection Significantly Increases Monocyte Transmigration Across the BBB in Response to CCL2. “Day 3” monocytes from 8 separate individuals were infected with HIV or remained uninfected as described in the methods, added to our BBB model, and allowed to transmigrate for 24 hours to media alone or to 200 ng/mL CCL2. (**A**) The total number of CD14^+^CD16^+^ monocytes that transmigrated to media (circles) or CCL2 (squares) was determined. (**B**) The pooled average fold change in monocyte transmigration to media (white bars, set to 1) or CCL2 (black bars), was determined. HIV infected CD14^+^CD16^+^ monocytes transmigrated exuberantly in response to CCL2. This did not occur in the absence of the chemokine in response to media alone. Data are represented as mean ± standard error of the mean. Significance was determined using the Wilcoxon Signed Rank test. *p<0.01.

We previously examined the transmigration of uninfected, CD14^+^CD16^+^ monocytes across our tissue culture model of the human BBB in response to CCL2 [17]. To characterize the effects of HIV infection on transmigration across the BBB, HIV infected and uninfected monocytes from 8 separate individuals were cultured nonadherently and added to our BBB model (Figure 2A-2B). CCL2 promoted significantly increased transmigration of uninfected CD14^+^CD16^+^ monocytes across the BBB compared to media alone. Upon HIV infection, there was an additional significant increase in CCL2-mediated transmigration. There was no difference in the baseline transmigration of HIV infected CD14^+^CD16^+^ monocytes when compared to uninfected cells (Figure 2A-2B). This indicates that the increase in transmigration of HIV infected CD14^+^CD16^+^ monocytes is CCL2 specific and does not occur in the absence of the chemokine. The CCL2-mediated increase in transmigration of HIV infected monocytes was not dependent on the extent of viral replication and occurred even at very low levels of infection. Secreted HIV p24 levels in the medium were 2462 ± 2567 pg/mL (n=31). Our findings indicate that HIV infected CD14^+^CD16^+^ monocytes may continually enter the CNS even with successful cART when virus is low to undetectable.

### HIV Infected Monocytes Are Highly Sensitive to CCL2

**Figure 3:**
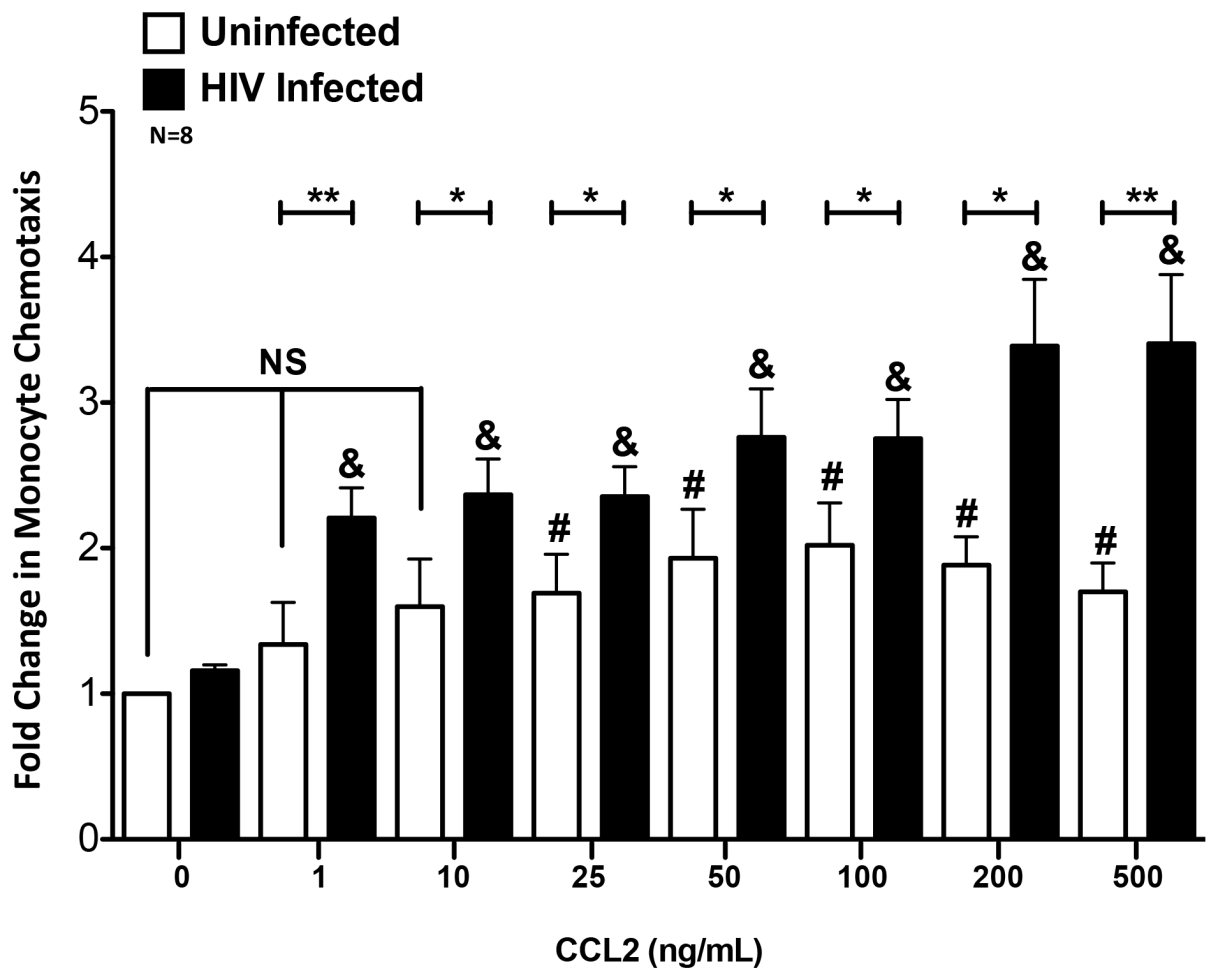
HIV Infected Monocytes Have Highly Increased Sensitivity to CCL2. Chemotaxis across a 5 µm polycarbonate membrane to increasing concentrations of CCL2 ranging from 1–500 ng/mL was examined in uninfected and HIV infected monocytes derived from eight independent donors. HIV infection (black bars) significantly increased monocyte chemotaxis to CCL2 relative to uninfected cells (white bars) at all concentrations. Uninfected cells did not chemotax to concentrations below 25 ng/mL, whereas HIV infected cells migrated even to very low levels of CCL2 (1 and 10 ng/mL). Data are represented as mean ± standard error of the mean. Significance was determined using a Wilcoxon Signed Rank test. ^#^p<0.05 indicates significant chemotaxis relative to 0 ng/mL CCL2 for the uninfected cells. ^&^p<0.01 indicates significant chemotaxis relative to 0 ng/mL CCL2 for the HIV infected cells. ^*^p<0.05 and **p<0.01 indicate significant chemotaxis of HIV infected cells relative to uninfected cells. NS indicates no significant change as compared to 0 ng/mL CCL2 for the uninfected cells.

To characterize further the mechanisms by which CCL2 and HIV infection increased monocyte transmigration, we performed chemotaxis assays with a microchemotaxis chamber rather than our BBB model to assess the direct effect of HIV infection and this chemokine on monocytes. Uninfected and HIV infected monocytes from our culture system were examined for chemotaxis across a polycarbonate membrane to diluent or to CCL2 concentrations ranging from 1–500 ng/mL (Figure 3). Uninfected monocytes exhibited a chemotactic dose response with 3 main features: 1) the lowest concentrations of CCL2 (1 and 10 ng/mL) failed to promote chemotaxis, 2) significant chemotaxis above baseline occurred at 25 ng/mL and increased with additional chemokine until it reached a maximum at 100 ng/mL, and 3) chemotaxis remained constant at the highest concentrations of CCL2, rather than increasing. In contrast, HIV infected monocytes migrated in numbers significantly higher than baseline to all concentrations of CCL2 (Figure 3). Unlike the uninfected cells, the response of HIV infected monocytes did not plateau even at the highest concentrations of CCL2, but continued to steadily increase. HIV infected monocytes migrated in greater numbers than the uninfected cells at all concentrations of CCL2, even at concentrations as low as 1 and 10 ng/mL, which failed to promote chemotaxis of the uninfected monocytes. This suggests that HIV infection increases the sensitivity of CD14^+^CD16^+^ monocytes to CCL2, enabling the infected cells to enter the CNS when CCL2 is at such low levels that normally would not elicit transmigration.

### HIV Infection Increases CCR2 on CD14^+^CD16^+^ Monocytes

**Figure 4:**
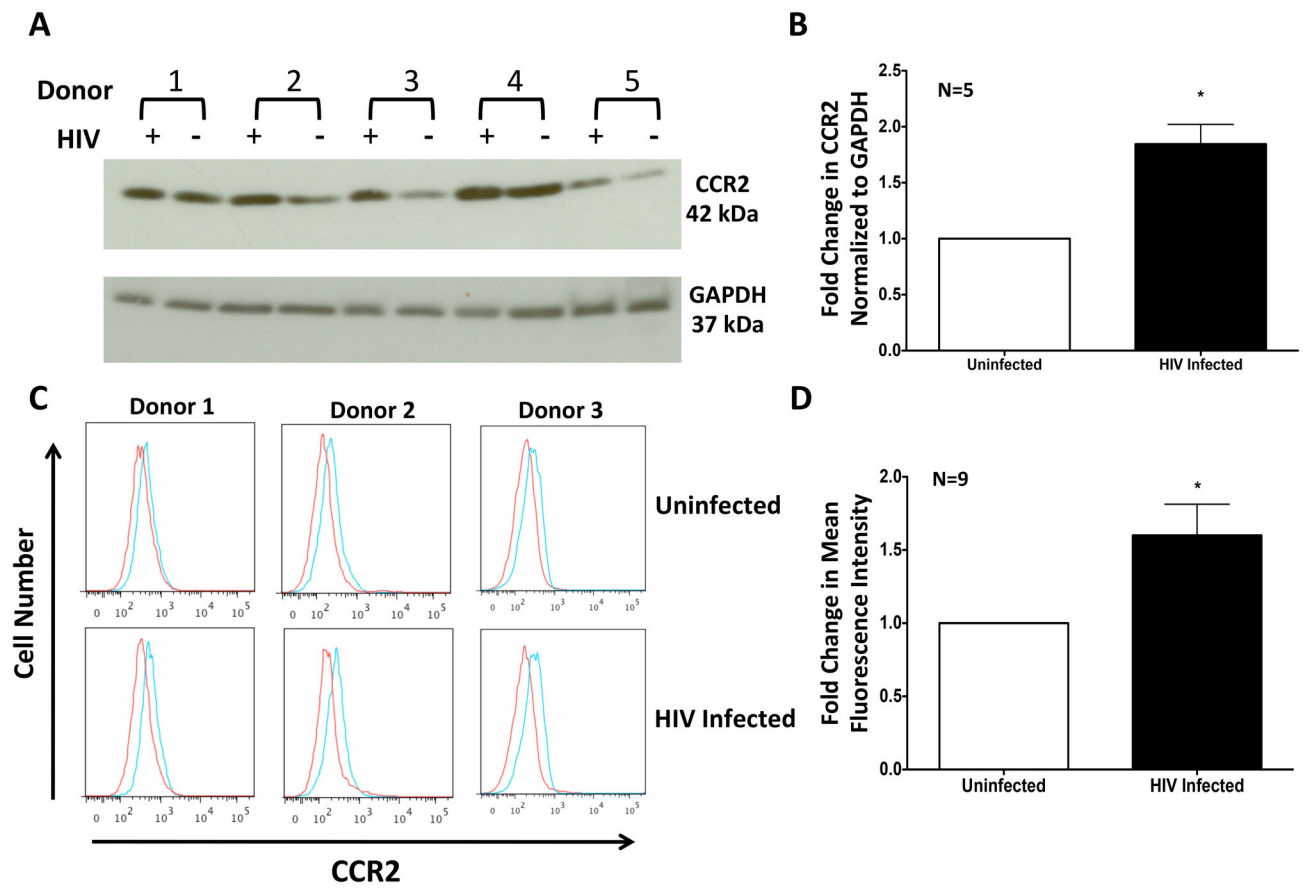
HIV Infection Increases Total and Surface Expression of CCR2 on CD14^+^CD16^+^ Monocytes. (**A**) HIV infected and uninfected monocytes from 5 independent individuals, enriched for the CD14^+^CD16^+^ population, were examined for CCR2 by Western blot. HIV infection increased total CCR2 within monocytes. (**B**) Densitometric analysis was performed and the average pooled fold change of CCR2 normalized to GAPDH was determined for uninfected (white bar) and HIV infected (black bar) monocytes. Data are represented as mean ± standard error of the mean. Significance was determined using a Two-Tailed Paired T test. *p<0.01. (**C**) HIV infected and uninfected monocytes were analyzed by flow cytometry using antibodies specific to CCR2 (blue) or an isotype matched negative control (red). Representative histograms of the CD14^+^CD16^+^ monocytes from three separate individuals are shown. (**D**) After subtracting the fluorescence contribution from the isotype matched control, the average fold increase of CCR2 on the surface of HIV infected (black bar) CD14^+^CD16^+^ monocytes relative to uninfected cells (white bar, set to 1) was 1.6 fold. Data are represented as mean ± standard error of the mean. Significance was determined using Wilcoxon Signed Rank test. *p<0.05.

To determine the mechanism that mediated the increased sensitivity of HIV infected CD14^+^CD16^+^ monocytes to CCL2, Western blot and FACS analyses were performed to quantify CCR2, the only known receptor for CCL2, on monocytes [22]. Monocytes from 5 separate individuals were isolated, cultured nonadherently, and infected with HIV_ADA_ or remained uninfected. We performed Western blot analysis on whole cell lysates from these cells (Figure 4A-4B). Regardless of the initial amount of CCR2 in monocytes from each individual, HIV infection significantly increased total CCR2.

To determine whether the increase in CCR2 occurred on the surface, where the receptor binds its ligand, monocytes were stained with antibodies specific for CD14, CD16, and CCR2 and analyzed by flow cytometry. We previously found that CD14^+^CD16^+^ monocytes expressed surface CCR2 [17,23]. HIV infection significantly increased CCR2 on the surface of CD14^+^CD16^+^ monocytes (Figure 4C-4D), as compared to uninfected cells. This suggests that the heightened sensitivity of HIV infected CD14^+^CD16^+^ monocytes to CCL2 occurs, at least in part, due to increased CCR2.

### Monocyte Maturation Increases Surface Junctional Proteins

**Figure 5:**
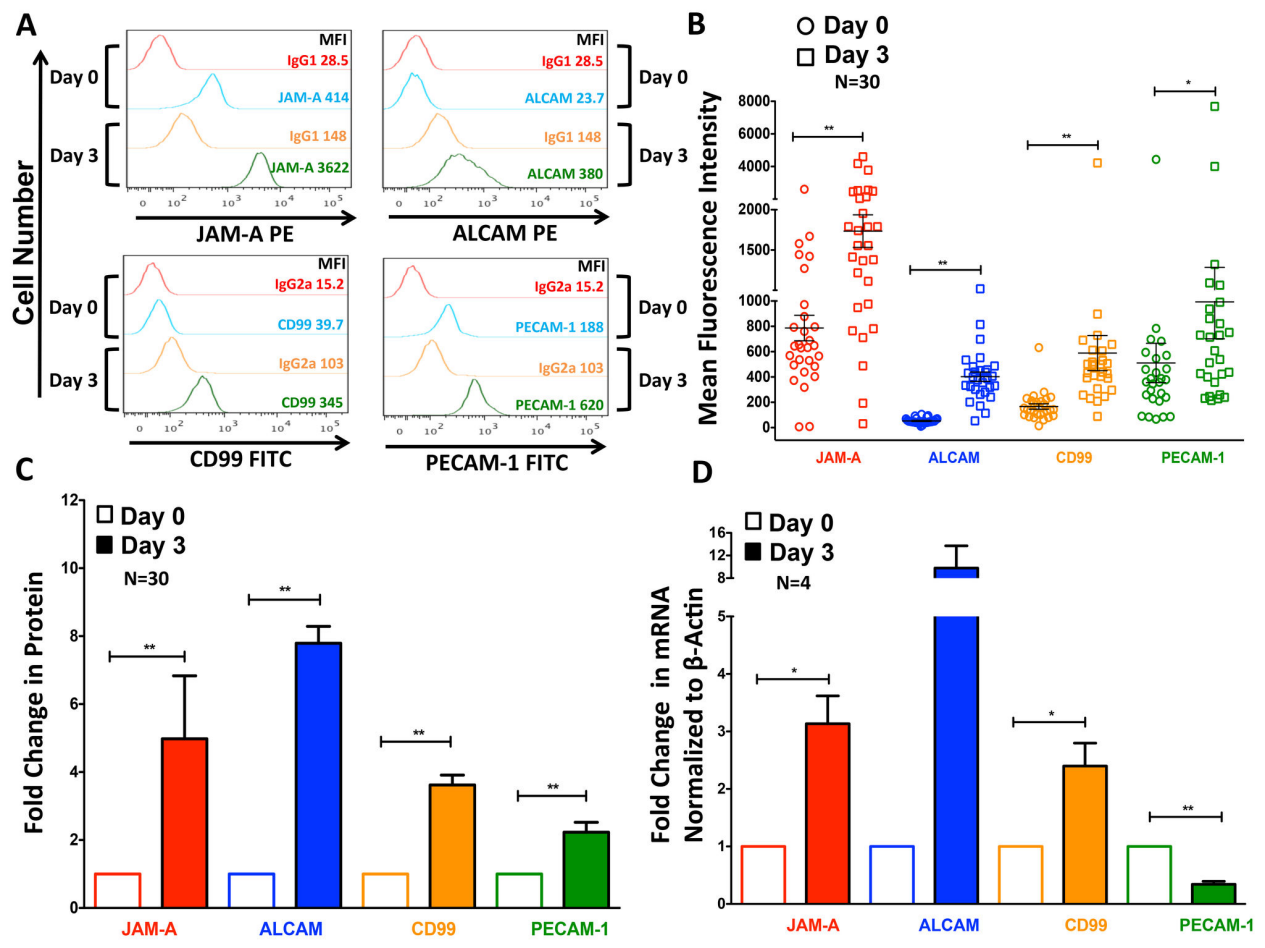
Surface Expression of Junctional Proteins Increases upon Monocyte Maturation. The surface expression of JAM-A, ALCAM, CD99, and PECAM-1 was analyzed by flow cytometry on freshly isolated, “Day 0” and “Day 3” monocytes. (**A**) FACS data represented as histograms show the surface junctional protein expression from one representative HIV(-) individual. The mean fluorescent intensity (MFI) of each protein, or the MFI obtained with each isotype matched negative control antibody, is indicated on “Day 0” and “Day 3”. There was an increase in each junctional protein as the monocytes matured from “Day 0” (blue) to “Day 3” (green). (**B**) After subtracting the contribution of the isotype matched negative control antibodies, the MFI of JAM-A (red), ALCAM (blue), CD99 (yellow), and PECAM-1 (green) were analyzed on monocytes from 30 different HIV(-) individuals on “Day 0” (circles) and “Day 3” (squares). The extent to which each junctional protein increased after nonadherent culture for 3 days varied among donors. (**C**) The fold increase in the MFI of each junctional protein on “Day 3” monocytes (shaded bars) from 30 individual HIV(-) donors relative to “Day 0” (white bars, set to 1) was determined. (**D**) qRT-PCR was performed to determine the mRNA expression of junctional proteins. RNA was isolated from “Day 0” and “Day 3” monocytes from 4 independent donors. The fold change on “Day 3” (solid bars) relative to “Day 0” (white bars, set to 1) of JAM-A (red), ALCAM (blue), CD99 (yellow), and PECAM-1 (green) normalized to β-Actin was determined. Data are represented as mean ± standard error of the mean. Significance was determined using Wilcoxon Signed Rank test or a Two-Tailed Paired T test. ^*^p<0.05**p<0.01. ALCAM was not significant due to high inter-donor variability, despite the large increases in mRNA expression (p=0.1).

Several surface proteins have been implicated in facilitating monocyte entry into peripheral tissues, but those specific to promoting entry into the brain parenchyma have not been fully characterized. It is important to have a more complete understanding of these surface markers on the CD14^+^CD16^+^ monocytes that enter the CNS in high numbers upon HIV infection. Dysregulation of these proteins may compromise the well-controlled entry of these mature monocytes across the BBB. We characterized JAM-A, ALCAM, CD99, and PECAM-1 on monocytes from 30 individuals after initial isolation (Day 0) and after maturation in our culture system (Day 3). These junctional proteins facilitate transendothelial migration in the periphery and we proposed would contribute to monocyte entry into the CNS during HIV infection [23]. JAM-A and PECAM-1 were expressed on the cell surface of freshly isolated monocytes, while there was minimal to no ALCAM or CD99 (Figure **5A**, blue lines compared to red lines). After enriching the CD14^+^CD16^+^ subset during three days of nonadherent culture, there was an increase in each junctional protein (Figure **5A** green lines compared to blue lines). This increase was consistent among 30 donors, although its extent varied (Figure 5B). Surface junctional proteins increased 2-8 fold on “Day 3” monocytes relative to freshly isolated cells (Figure 5C). Importantly, when we analyzed the junctional proteins on the surface of the 5-10% of the CD14^+^CD16^+^ population present in freshly isolated monocytes from uninfected people, the expression of these proteins was similar to that on monocytes cultured nonadherently for three days (not shown).

To characterize the mechanisms that mediated the increase in each junctional protein, qRT-PCR was performed on RNA isolated from monocytes before and after culture from 4 separate individuals (Figure 5D). With the exception of PECAM-1, there was an increase in the mRNA for each protein from “Day 0” to “Day 3” (Figure 5D) normalized to β-actin. Similar results were obtained when normalized to GAPDH (not shown). Inter-donor variability also existed at the gene expression level, particularly for ALCAM, for which 4 individual donors showed a 1.4, 7.3, 10.3, and 20.5 fold increase in ALCAM mRNA after culture as compared to freshly isolated cells (Figure **5D**, blue bars). The increase in PECAM-1 was not consistent at the mRNA level (Figure **5D**, green bars). The mechanisms by which PECAM-1 is increased on the surface after three days of nonadherent culture are unclear, and may occur by changes in localization or protein stability. We are pursuing these mechanisms in future studies.

### JAM-A and ALCAM Mediate Transmigration of CD14^+^CD16^+^ Monocytes Across the BBB

**Figure 6:**
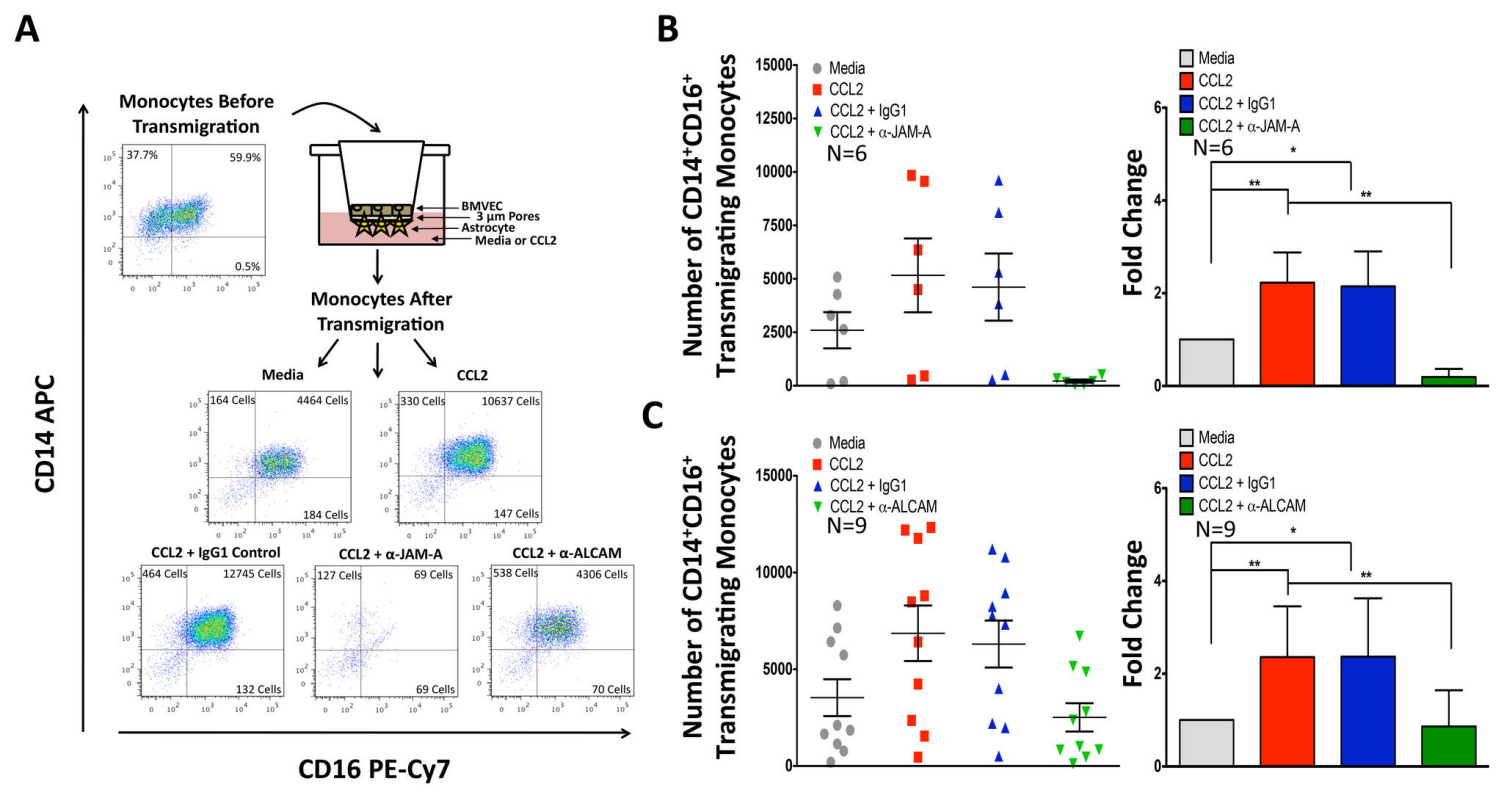
JAM-A and ALCAM are Critical for Transmigration of CD14^+^CD16^+^ Monocytes Across the BBB. “Day 3” monocytes highly enriched for CD14^+^CD16^+^ cells were added to our *in vitro* model of the human BBB in the presence of blocking antibodies to JAM-A, ALCAM or irrelevant isotype matched negative control antibody and allowed to transmigrate for 24 hours to 200 ng/mL CCL2. Monocytes were stained with CD14 APC and CD16 PE-Cy7-coupled antibodies. (**A**) Dot plots show the transmigration assay for a representative individual. Before transmigration, the “Day 3” monocytes consisted of both CD14^+^CD16^-^ and CD14^+^CD16^+^ cells. After transmigration across our human BBB model, the population consisted primarily of CD14^+^CD16^+^ cells. (**B**–**C**) Transmigration assays with monocytes from at least 6 independent individuals were performed and the number of CD14^+^CD16^+^ monocytes that transmigrated was quantified by FACS (**B–C left**). Depicts the total number of CD14^+^CD16^+^ monocytes that transmigrated for each experiment (**B–C, right**). Shows the average fold change in monocyte transmigration, relative to the baseline transmigration that occurred to media alone. CCL2 (red) promoted significantly increased transmigration compared to media alone (grey). The CCL2-mediated increase in transmigration was completely inhibited by antibodies (green) to JAM-A (**B**) or ALCAM (**C**). An isotype matched negative control antibody (blue) had no affect on transmigration. Data are represented as mean ± standard error of the mean. Significance was determined using Wilcoxon Signed Rank test. ^*^p<0.05**p<0.01.

To determine the role of the increased junctional proteins on “Day 3” monocytes in mediating entry into the CNS, we performed transmigration assays using our human BBB model. As we showed previously [17] and demonstrate in Figure **6**, the CD14^+^CD16^+^ monocyte population preferentially transmigrated across this model (Figure **6A**, CD14^+^CD16^+^ monocyte numbers before transmigration compared to those after transmigration) in response to CCL2 (Figure 6A–6C). Blocking JAM-A (Figure 6A-6B) and ALCAM (Figure 6A, 6C) with antibody decreased CCL2-mediated transmigration to baseline or below. There was no difference in transmigration to CCL2 in the presence of an isotype matched negative control antibody (Figure 6A–6C). This demonstrates the importance of these junctional proteins in facilitating monocyte transmigration across the BBB. Blocking experiments with antibodies to PECAM-1 and CD99 were inconclusive as the control antibodies also reduced transmigration.

### JAM-A and ALCAM Also Mediate Transmigration of HIV Infected CD14^+^CD16^+^ Monocytes Across the BBB

**Figure 7:**
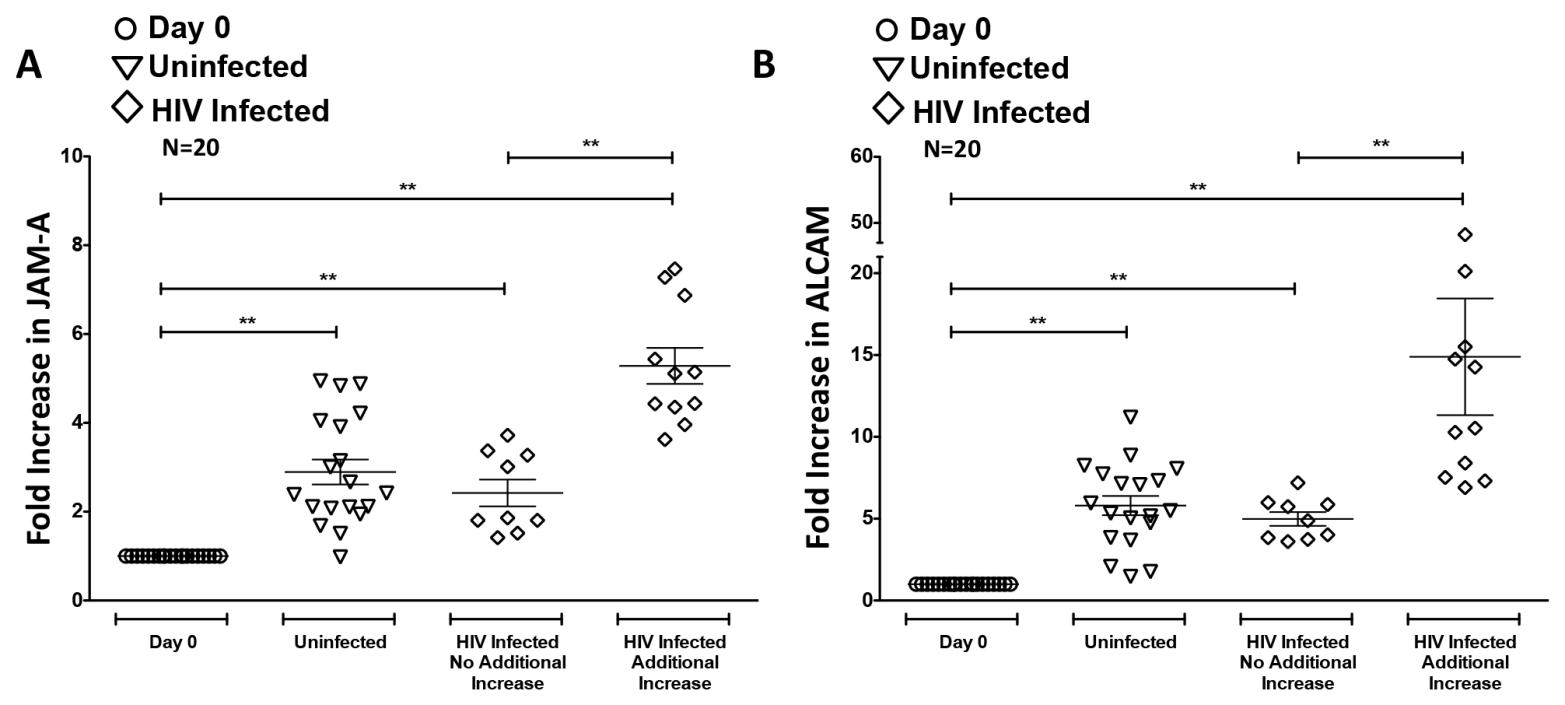
JAM-A and ALCAM Increase on CD14^+^CD16^+^ Monocytes Upon HIV Infection. The surface expression of JAM-A (**A**) and ALCAM (**B**) was analyzed by flow cytometry on HIV infected or uninfected monocytes from 20 independent individuals. After subtracting the contribution of the isotype matched negative control antibodies, the fold increase in the MFI of each junctional protein on the monocytes that were cultured nonadherently and were either HIV infected (diamonds) or remained uninfected (triangles), relative to the freshly isolated monocytes (circles, set to 1) was determined. The majority of the individuals (11/20) had an additional increase in JAM-A and ALCAM after HIV infection over that which had already occurred during monocyte maturation (see Figure 5). Monocytes from the same 11 individuals had an increase in both JAM-A and ALCAM. Data are represented as mean ± standard error of the mean. Significance was determined using Wilcoxon Signed Rank test. **p<0.01.

To determine the effect of HIV infection on JAM-A, ALCAM, CD99, and PECAM-1, monocytes from 20 independent people were isolated, cultured nonadherently for 3 days, and infected with HIV_ADA_ or remained uninfected. There was an increase in JAM-A (Figure 7A) and ALCAM (Figure 7B) as the monocytes matured in our culture system, as compared to “Day 0” expression that was set to 1. After HIV infection, there was an additional increase in surface JAM-A (Figure **7A**, HIV infected additional increase) and ALCAM (Figure **7B**, HIV infected additional increase) above that which had already occurred during monocyte maturation (compare to Figure 5) on monocytes isolated from 11 of the 20 individuals. The increase in the junctional proteins varied among donors, and ranged from 1.2–7 fold above that of the uninfected monocytes. There was no further increase in CD99 or PECAM-1 after HIV infection (not shown).

**Figure 8:**
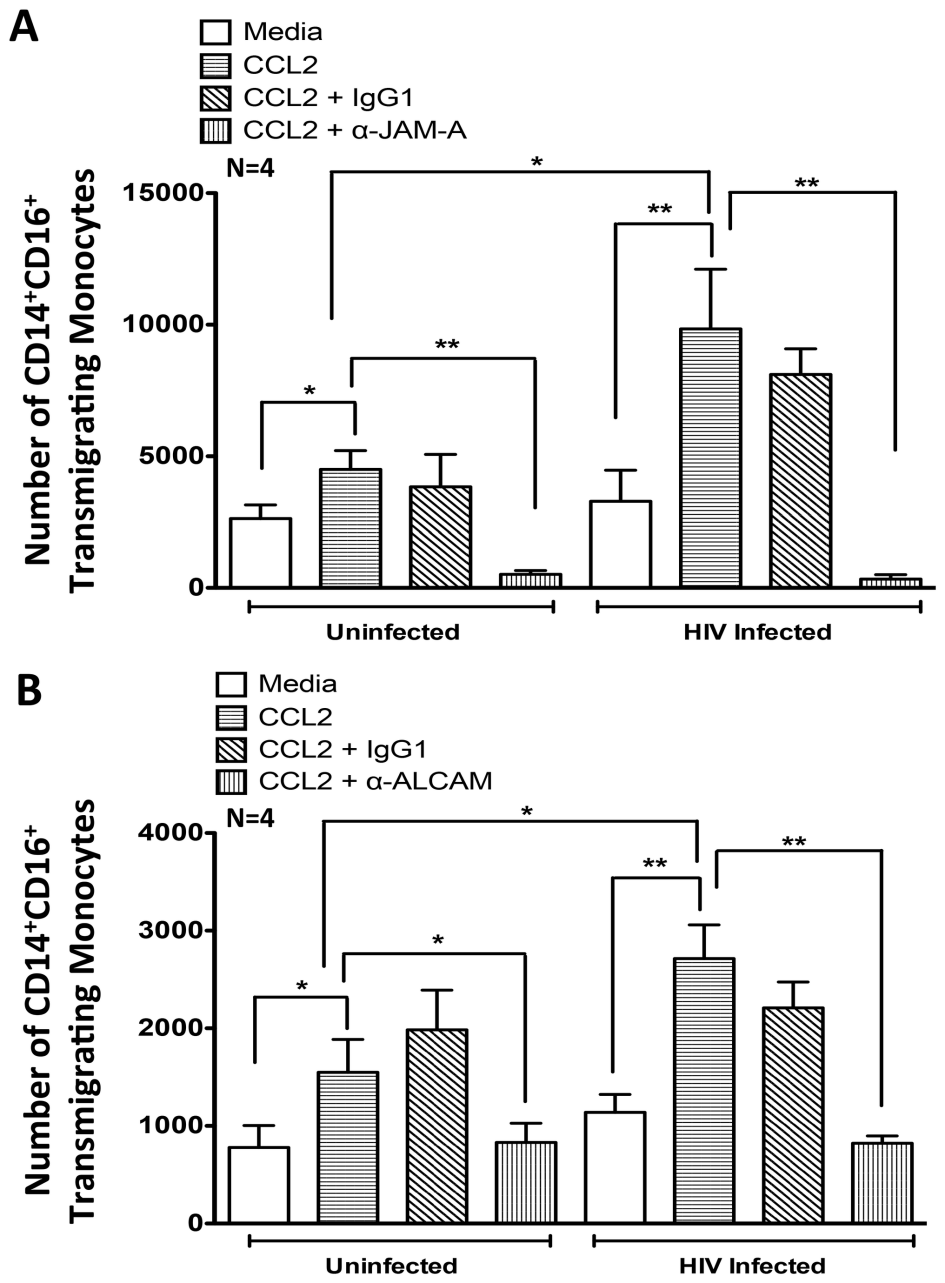
Blocking JAM-A and ALCAM Inhibits CCL2-Mediated Increase in HIV Infected Monocyte Transmigration Across the BBB. HIV infected or uninfected monocytes from 4 independent individuals were added to the BBB model and transmigrated in response to CCL2 in the presence of blocking antibodies to (**A**) JAM-A or (**B**) ALCAM. The number of CD14^+^CD16^+^ monocytes that transmigrated was analyzed by flow cytometry. CCL2 (horizontal hatching) promoted increased transmigration compared to media alone (white bars) for both uninfected and HIV infected cells. (**A**–**B**) HIV infected monocytes transmigrated in significantly greater numbers relative to uninfected monocytes in response to CCL2. Blocking antibodies (vertical hatching) to (**A**) JAM-A and (**B**) ALCAM decreased monocyte transmigration similarly for both HIV infected and uninfected monocytes. An isotype matched negative control antibody (diagonal hatching) did not affect CCL2-mediated transmigration. Data are represented as mean ± standard error of the mean. Significance was determined using a Two-Tailed Paired T test. ^*^p<0.05**p<0.01.

To examine the contribution of increased JAM-A and ALCAM on the surface of HIV infected CD14^+^CD16^+^ monocytes, we performed transmigration assays in the presence of antibodies to each junctional protein. Despite the high numbers of HIV infected CD14^+^CD16^+^ monocytes transmigrating across the BBB in response to CCL2 (Figure 2), antibodies to JAM-A (Figure 8A) and ALCAM (Figure 8B) decreased transmigration of infected cells to a similar extent as for the uninfected cells (Figure **6** Figure 8). The antibodies to JAM-A and ALCAM blocked monocyte transmigration even for the individuals whose cells had an additional increase in the junctional proteins upon HIV infection.

## Discussion

In this study, we demonstrated that HIV infected CD14^+^CD16^+^ monocytes had a heightened sensitivity to CCL2 mediated by increased surface CCR2 and transmigrated in greater numbers across our model of the human BBB in response to CCL2 than did uninfected cells. This process may be facilitated by increased JAM-A, ALCAM, CD99, and PECAM-1 on CD14^+^CD16^+^ monocytes relative to their CD14^+^CD16^-^ counterparts. At least two of these junctional proteins, JAM-A and ALCAM, were important for the transmigration of both HIV infected and uninfected CD14^+^CD16^+^ monocytes across the BBB in response to CCL2 as blocking antibodies completely inhibited this process.

The CD14^+^CD16^+^ monocyte population is critical to the establishment of neuroAIDS [15–17,23]. We found that this mature monocyte subset was increased in the peripheral blood of HIV(+) individuals compared to HIV(-) people, despite cART, and that our culture system modeled this expansion. CD14^+^CD16^+^ monocytes are highly susceptible to infection with HIV [15–17,38]. Characterizing the effects of HIV infection on their transmigration across the BBB is key to the development of therapeutic strategies.

We found that HIV infected CD14^+^CD16^+^ monocytes crossed our BBB model in significantly higher numbers in response to CCL2 than did uninfected cells. The baseline transmigration of these HIV infected monocytes remained the same as that of uninfected cells, indicating that CCL2 must be present for the increased migration. It is important to note that there was very low-level infection of the CD14^+^CD16^+^ monocytes, yet there was still exuberant transmigration in response to CCL2. This indicates that high levels of transmigration are not dependent on viral load and would occur in HIV(+) individuals with well controlled virus due to cART. This models HIV infection in the cART era where viral loads are low to undetectable, and demonstrates that high numbers of latently infected CD14^+^CD16^+^ monocytes may continue to enter the CNS, despite successful therapy, mediating ongoing chronic neuroinflammation.

Using a chemotaxis assay, we found the exuberant response to CCL2 occurred due to increased sensitivity of HIV infected monocytes to the chemokine. HIV infected monocytes had a sustained, increased chemotactic response to CCL2 over a wide range of concentrations, including very low amounts of CCL2 that did not induce chemotaxis of uninfected cells. This suggests that early after peripheral infection, HIV infected CD14^+^CD16^+^ monocytes will enter the CNS in response to baseline levels of CCL2 constitutively present in the brain, providing a mechanism by which the initial seeding of the CNS with HIV occurs. The uninfected cells would not be primed to respond to such low levels of CCL2. After this initial insult, CCL2 becomes elevated in the CNS and CSF of HIV infected individuals [39,40]. During this phase both HIV infected and uninfected monocytes would enter the CNS, resulting in neuroinflammation and creating a viral reservoir.

This heightened sensitivity to CCL2 was mediated by increased CCR2 on HIV infected CD14^+^CD16^+^ monocytes. CCR2 on monocytes is downregulated with most inflammatory conditions *in vitro* and *in vivo*, including TNF-α or LPS treatment as well as in atherosclerotic plaques and multiple sclerosis lesions [41–43]. Despite being an inflammatory process, HIV infection promoted the upregulation or maintenance of CCR2 on CD14^+^CD16^+^ monocytes that primed hypersensitivity to CCL2 and enabled the preferential transmigration of infected cells across the BBB. CCR2 may represent an adjunctive target to reduce the neuroinflammation that occurs during HIV infection. CCR2 antagonists have been developed as potential therapeutics for many other inflammatory diseases [44].

As monocytes mature in the peripheral blood, they express markers associated with tissue macrophages [17]. In addition to CD16, some of these include CD163 and Mac387, proteins implicated in the pathogenesis of HIV/SIV [45,46]. Our studies suggest that increased surface JAM-A, ALCAM, CD99, and PECAM-1 represent additional monocyte maturation markers. These molecules may facilitate the preferential transmigration of CD14^+^CD16^+^ cells across the BBB. The average increase for all of the junctional proteins during monocyte maturation ranged from 2–8 fold, but there were individuals whose increase was as much as 15-49 fold. Additionally, we found that CD14^+^CD16^+^ monocytes isolated from 11 out of 20 individuals had a further increase in JAM-A and ALCAM upon HIV infection, as compared to the increase in the junctional proteins that had already occurred during monocyte maturation from “Day 0” to “Day 3”. The same 11 individuals whose monocytes had increased JAM-A upon HIV infection also had an increase in ALCAM, suggesting that host genetic factors predispose certain individuals to an HIV-mediated increase in junctional proteins.

Diapedesis is a highly regulated process during which homophilic interactions between the junctional proteins present on the monocyte interact with those on the BMVEC of the BBB to shepherd the monocyte into the CNS. The increase in surface junctional proteins on CD14^+^CD16^+^ monocytes during maturation and HIV infection may disrupt the normal interactions that occur between monocytes and the BBB required for controlled transmigration. Increased junctional proteins have been implicated in many pathologies. ALCAM is increased in cancers and is associated with metastasis, further suggesting that higher expression of this protein facilitates cell migration into tissue [47]. JAM-A and ALCAM are increased peripherally and in the brain during inflammation [48,49]. ALCAM is also increased on the BMVEC of the BBB in HIV(+) individuals who have histories of cocaine abuse [50].

During inflammation the junctional proteins may also be shed from the surface. These shed proteins may compete with the specific homophilic binding between monocytes and BMVEC or the interactions that occur between BMVEC critical for establishing BBB integrity. Shedding may promote unstable junctional protein interactions that result in BBB disruption and increased transmigration. We previously demonstrated that HIV(+) individuals had increased shed PECAM-1 in sera and brain tissue and that increased shedding occurred *in vitro* from HIV infected PBMC treated with CCL2 [51]. Shed ALCAM in the CSF was shown to correlate with decreased basal ganglia function of individuals with HAND [52]. The presence of increased and shed junctional proteins on monocytes and within the brain may compromise the CNS of HIV infected individuals.

We found that antibodies to JAM-A and ALCAM inhibited transmigration of HIV infected and uninfected CD14^+^CD16^+^ monocytes across the BBB. Blocking JAM-A and ALCAM may represent novel therapeutic strategies to prevent monocyte entry into the brain during HIV infection, although this must be approached with caution. Immune cell trafficking is important for the host response to pathogens and targeting host proteins to decrease inflammation requires careful consideration. The monoclonal antibody to α_4_ integrin, Natalizumab, inhibited entry of leukocytes into the CNS of individuals with multiple sclerosis and initially showed promise as a therapeutic. Natalizumab inhibited even baseline immune cell surveillance. This resulted in the reactivation of JC virus in some individuals who developed a fatal progressive multifocal leukoencephalopathy [53]. HIV infected people are highly susceptible to opportunistic infections within the CNS, and a complete blockade of monocyte trafficking may be deleterious. ALCAM represents an attractive target as antibodies to it reduced transmigration to baseline, but not below, suggesting that it would not interfere with basal immune cell trafficking into the CNS. We found that ALCAM was primarily present on CD14^+^CD16^+^ monocytes and was minimally expressed on CD14^+^CD16^-^ cells, indicating that anti-ALCAM therapeutics would preferentially target the mature monocyte population so critical to the neuropathogenesis of HIV. There is greater ALCAM expression on the BMVEC of the BBB as compared to peripheral endothelial cells, suggesting that targeting ALCAM may specifically prevent CNS entry with minimal affects on other organs [24].

NeuroAIDS continues to persist in 40-70% of HIV infected individuals even with successful cART. Transmigration of the CD14^+^CD16^+^ subpopulation, that is highly susceptible to HIV infection, across the BBB into the CNS is critical to the pathogenesis of HAND. Our studies characterize some of the mechanisms that contribute to the entry of this mature monocyte subset into the brain including a highly increased sensitivity to CCL2 upon HIV infection that is CCR2-mediated, as well as increased surface JAM-A and ALCAM. Our findings indicate therapeutic strategies that may decrease the entry of this mature monocyte population into the CNS of HIV infected individuals, contributing to the eradication of HAND and CNS viral reservoirs.
